# Supplementary material for: Leucine-rich repeat kinase 2 impairs the release sites of Parkinson’s disease vulnerable dopamine axons
Source: bioRxiv. 2025 Aug 28:2025.08.28.672006. Preprint. [Version 1] doi: 10.1101/2025.08.28.672006 (PMC12407872; doi:10.1101/2025.08.28.672006)
Supplement: Supplementary file 1 [file NIHPP2025.08.28.672006v1-supplement-1.pdf]

bioRxiv preprint doi: https://doi.org/10.1101/2025.08.28.672006; this version posted August 28, 2025. The copyright holder for this preprint (which was not certified by peer review) is the author/funder, who has granted bioRxiv a license to display the preprint in perpetuity. It is made available under aCC-BY 4.0 International license.

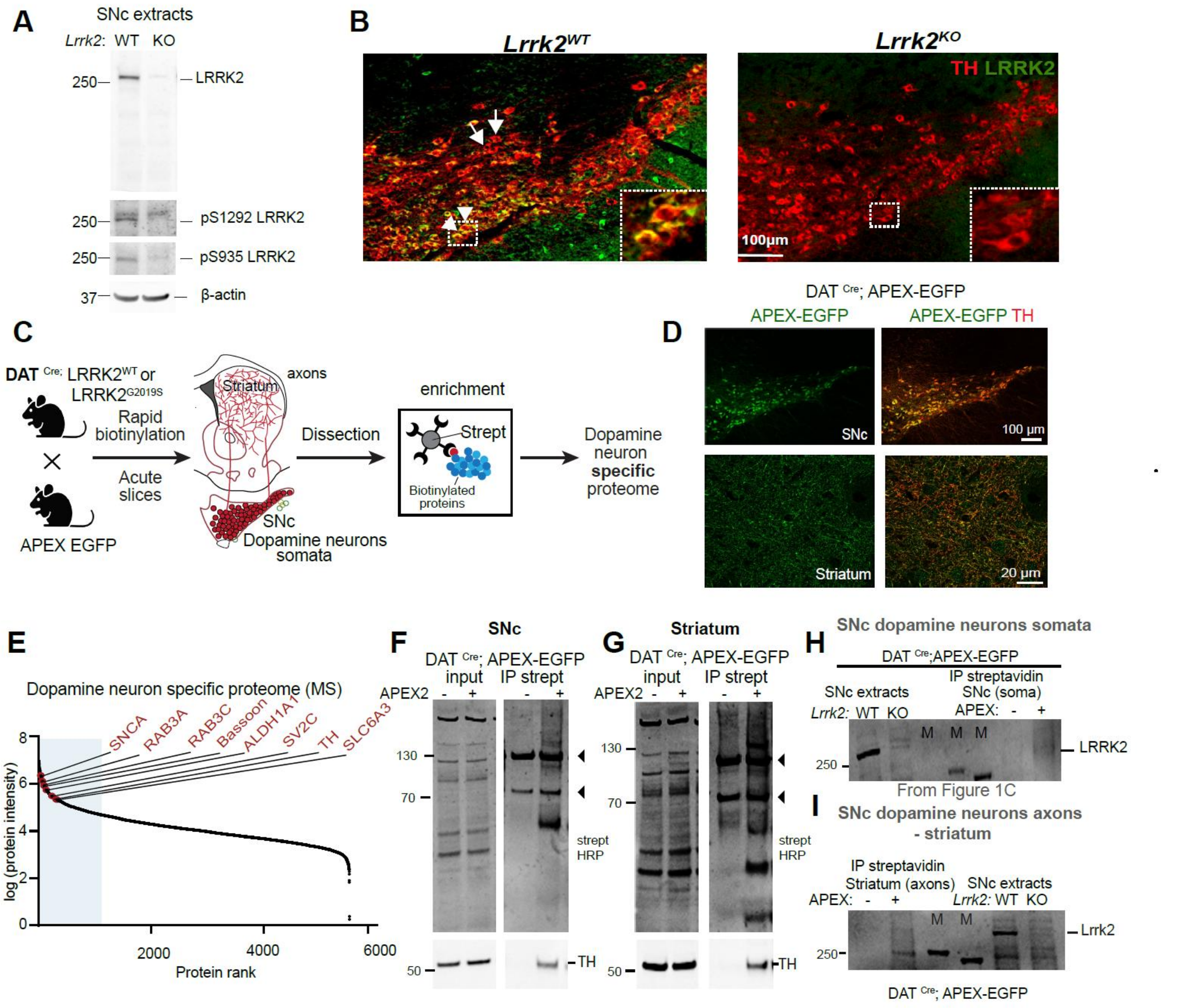

Supplementary Figure 1. LRRK2 protein is expressed in SNc dopamine neurons (linked to Figure 1). A. WB analysis of SNc brain extracts from *Lrrk2*^{WT} and *Lrrk2*^{KO} mice probed with the antibodies indicated on the right. Note the absence of the LRRK2 signal in the *Lrrk2*^{KO} tissues. **B.** Brain sections from *Lrrk2*^{WT} and *Lrrk2*^{KO} mice were stained with the LRRK2 antibody from A, along with TH. **C.** Workflow of APEX2-based proximity labeling within genetically labeled dopamine neurons in the mouse brain. Acute brain slices from DAT^{Cre}; LRRK2^{WT} and DAT^{Cre}; LRRK2^{G2019S} mice crossed with the APEX2 EGFP reporter mouse line[45] were rapidly biotinylated, quenched, and dissected. Tissues were lysed and precipitated to remove free biotin. Tissue proteins were subjected to streptavidin bead purification to enrich biotinylated proteins. On-bead digestion yielded peptides enriched from dopamine neurons, which were analyzed using mass spectrometry. **D.** Representative images showing the cre-dependent expression of EGFP reporter along with Th immunostaining in the SNc and striatum of a DAT^{Cre}; LRRK2^{WT}; APEX2 EGFP mouse. **E.** 85% of the top 55 mDA neuron marker genes (e.g., TH and DAT; SLC6A3) from a publicly available APEX2 proteome dataset (dataset identifier PXD026229 ProteomeXchange Consortium) were detected in all our mass spectrometry samples from DAT^{Cre}; APEX2 EGFP mice. 40% of these markers were among the top 20% (1105/5525) (shaded light blue) of the most highly expressed proteins by intensity. **F.** WB analysis of biotinylated protein inputs and their streptavidin pulldowns from dissections of the indicated brain regions probed with antibodies as indicated on the right. Input, 10 µg of total protein (~1% of striatal lysate, and ~5% of SNc); equal volume of eluted proteins was loaded in each lane. **G.** Full gel from experiment presented in Figure 1C showing SNc extracts from *Lrrk2*^{WT} and *Lrrk2*^{KO} mice for reference of LRRK2 protein size. **H.** WB showing streptavidin pulldowns from equal volumes of proteins from the striatum of DAT^{Cre}; APEX2 EGFP mice or Cre- controls; SNc lysates from *Lrrk2*^{WT} and *Lrrk2*^{KO} are used for reference for LRRK2 protein size. M, lanes loaded with molecular size markers.

bioRxiv preprint doi: https://doi.org/10.1101/2025.08.28.672006; this version posted August 28, 2025. The copyright holder for this preprint (which was not certified by peer review) is the author/funder, who has granted bioRxiv a license to display the preprint in perpetuity. It is made available under aCC-BY 4.0 International license.

[44]s.

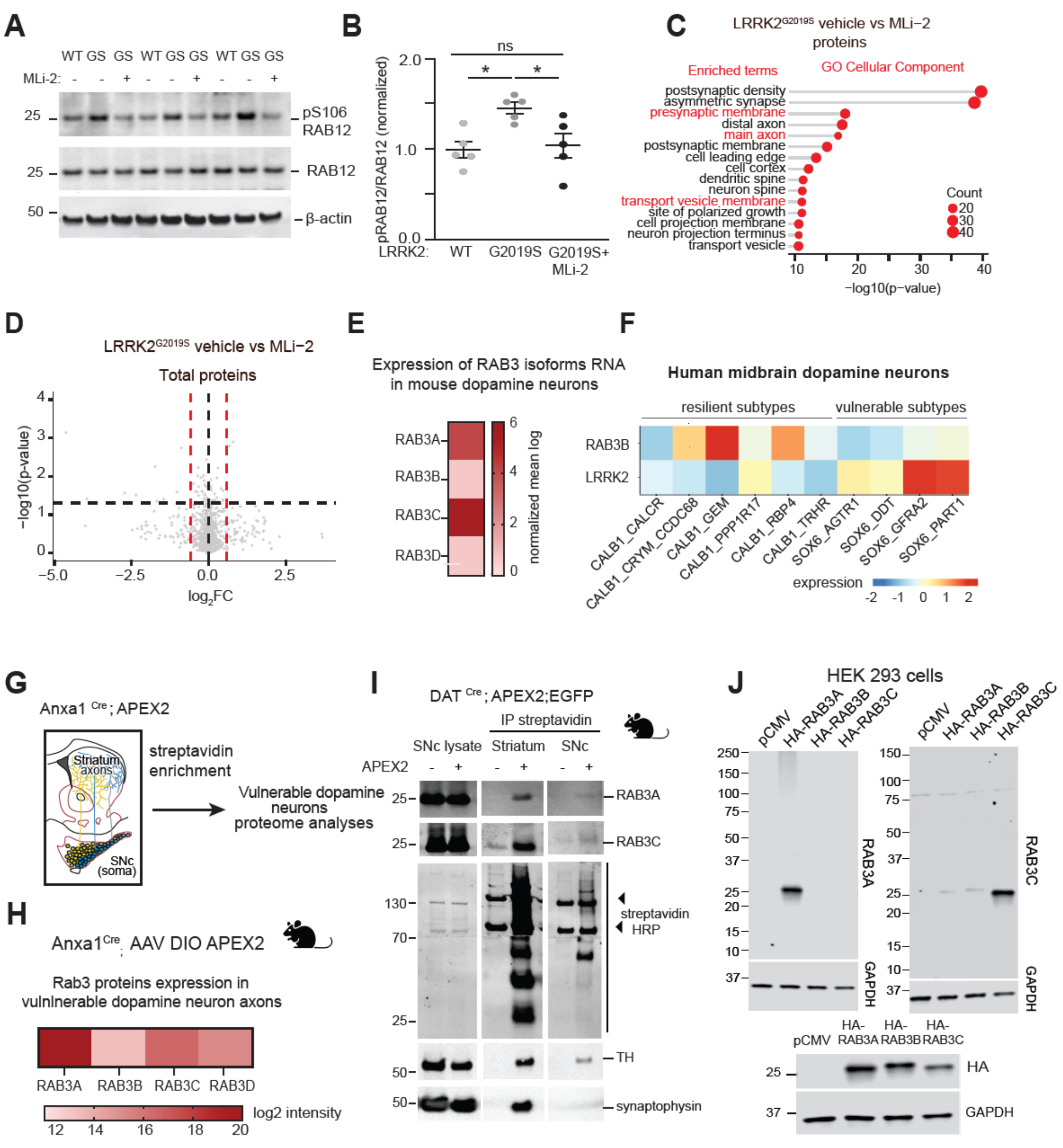

Supplementary Figure 2. Heterogeneous RAB3 isoform expression across dopamine neuron subtypes (linked to Figure 2). A. WB analysis of SNc extracts from mice previously treated with MLi-2 (10 mg/kg) or vehicle for 2 h and probed for pS106 RAB12 (LRRK2 kinase target), total RAB12, and β-actin. **B.** Quantification of p-RAB12 band intensities normalized to total RAB12. n=5 mice/condition. Data are represented as mean±SEM (error bars). Asterisks denote statistical significance for Šídák's multiple comparison tests after two-way ANOVA. *$p< 0.05$. (Treatment factor $F(1,12)=8.614$ $p=0.0125$, Genotype factor $F(1, 12)=10.64$, $p=0.0068$) **C.** Gene Ontology analysis of proteins with at least one differentially regulated phosphopeptide in striatal synaptosomes from vehicle vs MLi2-treated LRRK2^{G2019S} mice. The top 15 pathways significantly enriched in the cellular component term analysis (adjusted *p*-value ≤ 0.05 by hypergeometric test with HB correction). All enriched pathways have been uploaded to Zenodo. Link in the resources table.**D.** Volcano plot comparing the significantly altered total proteins (|Log2FC| > 0.58 and unadjusted *p*-value ≤ 0.05 by multiple unpaired t-tests) between vehicle and MLi-2-treated LRRK2^{G2019S} striatal synaptosomes. **E**. Heat map of the expression of RAB3 isoforms in a dataset of single cell RNA sequencing.[9] The differential expression of RAB3 isoforms in TH+ SNc neurons compared to the remaining non-TH SNc neurons is expressed as normalized mean log values

bioRxiv preprint doi: https://doi.org/10.1101/2025.08.28.672006; this version posted August 28, 2025. The copyright holder for this preprint (which was not certified by peer review) is the author/funder, who has granted bioRxiv a license to display the preprint in perpetuity. It is made available under aCC-BY 4.0 International license.

(RAB3A = 4.11, RAB3B = 0.693, RAB3C = 5.59, and RAB3D = 0.693). **F.** Heat map illustrating the expression of LRRK2 and RAB3B isoform in human dopamine neuron subpopulations from a single nucleus RNA sequencing dataset,[49] plotted with Shinny Cell.[931] **G.** Workflow of APEX2 experiment for dopamine neuron subpopulation-specific analysis with subcellular compartment resolution. Cre-dependent APEX2 expressing AAV (AAV5-CAG-DIO-APEX2-NES) was injected into the SNc of Anxa1^{Cre} LRRK2^{WT} mice for Anxa1+ dopamine neuron-specific APEX2 labeling. **H.** Relative expression of RAB3 protein isoforms in our Anxa1+ dopamine axon subcluster proteomic dataset (for further details see Supplementary Figure 6)**. I.** Equal amounts of eluted proteins from streptavidin pulldowns from the striatum and SNc from either a LRRK2^{WT} or a LRRK2^{G2019S} DAT^{Cre} mouse injected with APEX2 AAV**. J.** HEK293T cells were transiently transfected with either pCMV or HA-tagged RAB3 isoforms and blotted with isoform-specific antibodies or anti-HA, and with GAPDH for loading control. Equal expression of all isoforms is shown in the panel below.

bioRxiv preprint doi: https://doi.org/10.1101/2025.08.28.672006; this version posted August 28, 2025. The copyright holder for this preprint (which was not certified by peer review) is the author/funder, who has granted bioRxiv a license to display the preprint in perpetuity. It is made available under aCC-BY 4.0 International license.

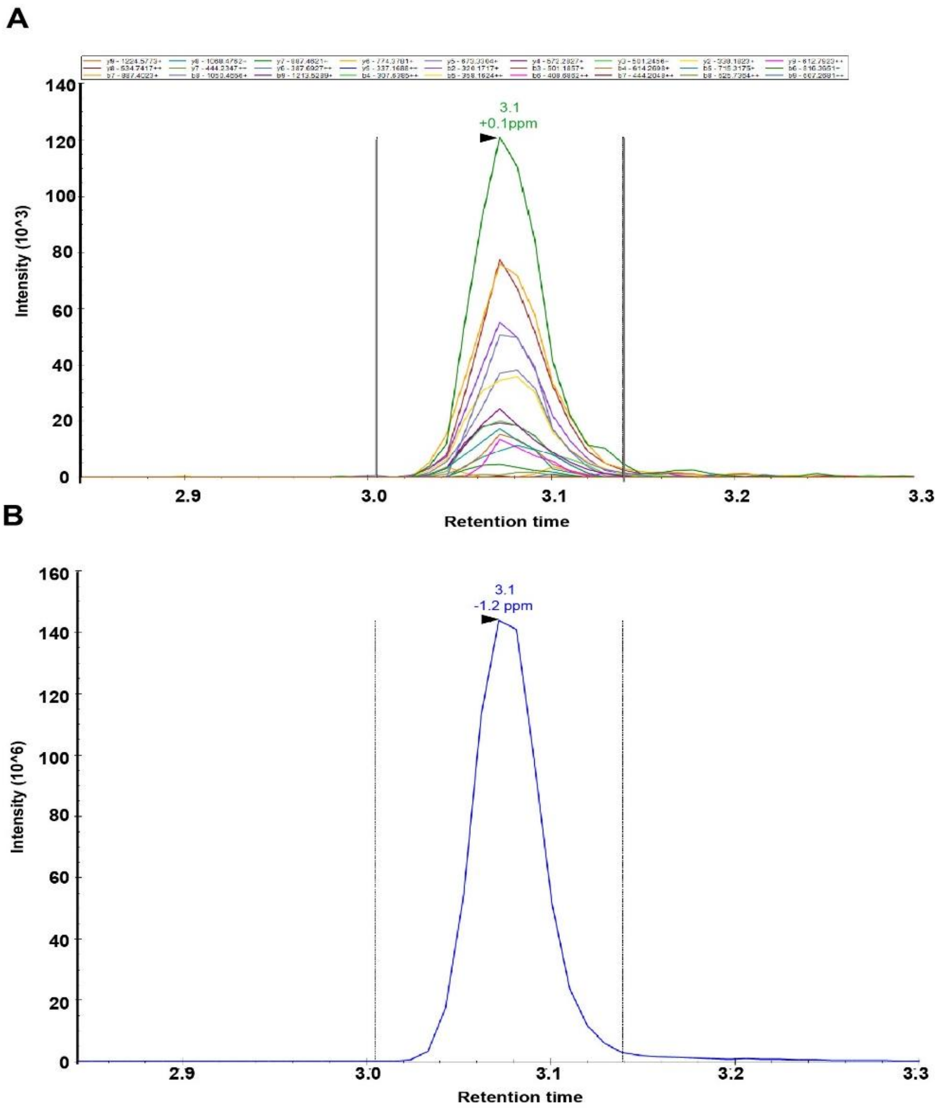

Supplementary Figure 3. Differential interactome of p-RAB3A and unphosphorylated RAB3A in extracts from mouse brain (linked to Figure 3).

A. Peptide fragment ions of the phosphopeptide; YRTITTAYR, of the 2+ precursor ion **(B).** which spans phosphorylation site T86 in recombinant p-Flag-RAB3A protein. All chromatograms elute at the same retention time, with peptide identification at a false-discovery rate of <1% (DIA-NN 1.9.2).

bioRxiv preprint doi: https://doi.org/10.1101/2025.08.28.672006; this version posted August 28, 2025. The copyright holder for this preprint (which was not certified by peer review) is the author/funder, who has granted bioRxiv a license to display the preprint in perpetuity. It is made available under aCC-BY 4.0 International license.

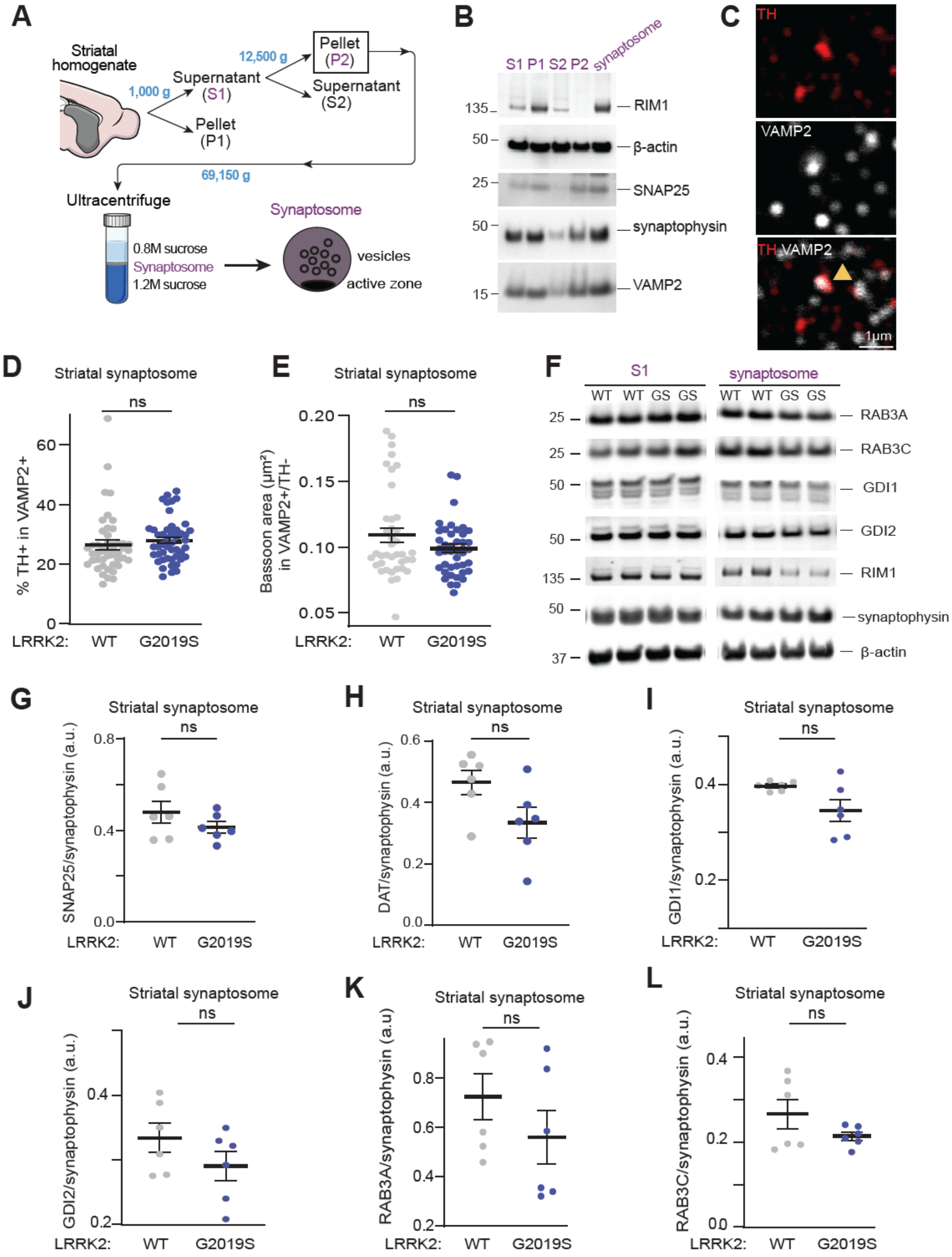

Supplementary Figure 4. LRRK2 alters the distribution and organization of active zones in striatal synaptosomes (linked to Figure 4). A. Schematic representation of the steps for striatal synaptosome fraction preparation**. B.** WB analysis of selective fractions from striatal subcellular fractionation, probed with antibodies as indicated on the right. This analysis shows enrichment of synaptic vesicle and active zone proteins. **C.** Confocal image of striatal synaptosomes stained with TH and VAMP2. A double positive synaptosome is indicated with the arrowhead. **D.** Quantification of the percentage of TH+/VAMP2+ striatal synaptosomes in LRRK2^{WT} and LRRK2^{G2019S} mice (denoted by WT and GS, respectively). Each circle represents the average result of an area with around 1,000 synaptosomes. n = 10-14 areas/4 mice per genotype. **E.** Quantification of bassoon area in VAMP2+ but TH- synaptosomes. Each circle represents the average of an image with ~1,000 synaptosomes. n=10-14 areas/4 mice per genotype. Data in D and E represent mean± SEM. Ns denotes no statistical significance after unpaired t-tests. **F.** WB analysis of S1 and synaptosome fraction from LRRK2^{WT} and LRRK2^{G2019S} mice using the antibodies shown on the right. **G-L**. Quantification of the indicated protein signals in striatal synaptosomes from LRRK2^{WT} and LRRK2^{G2019S}. Data are mean± SEM. Ns =non-significant differences in comparisons after unpaired t-tests. n= 6 mice/genotype. Equal amounts of proteins were loaded in all experiments, and duplicates represent biological replicates.

bioRxiv preprint doi: https://doi.org/10.1101/2025.08.28.672006; this version posted August 28, 2025. The copyright holder for this preprint (which was not certified by peer review) is the author/funder, who has granted bioRxiv a license to display the preprint in perpetuity. It is made available under aCC-BY 4.0 International license.

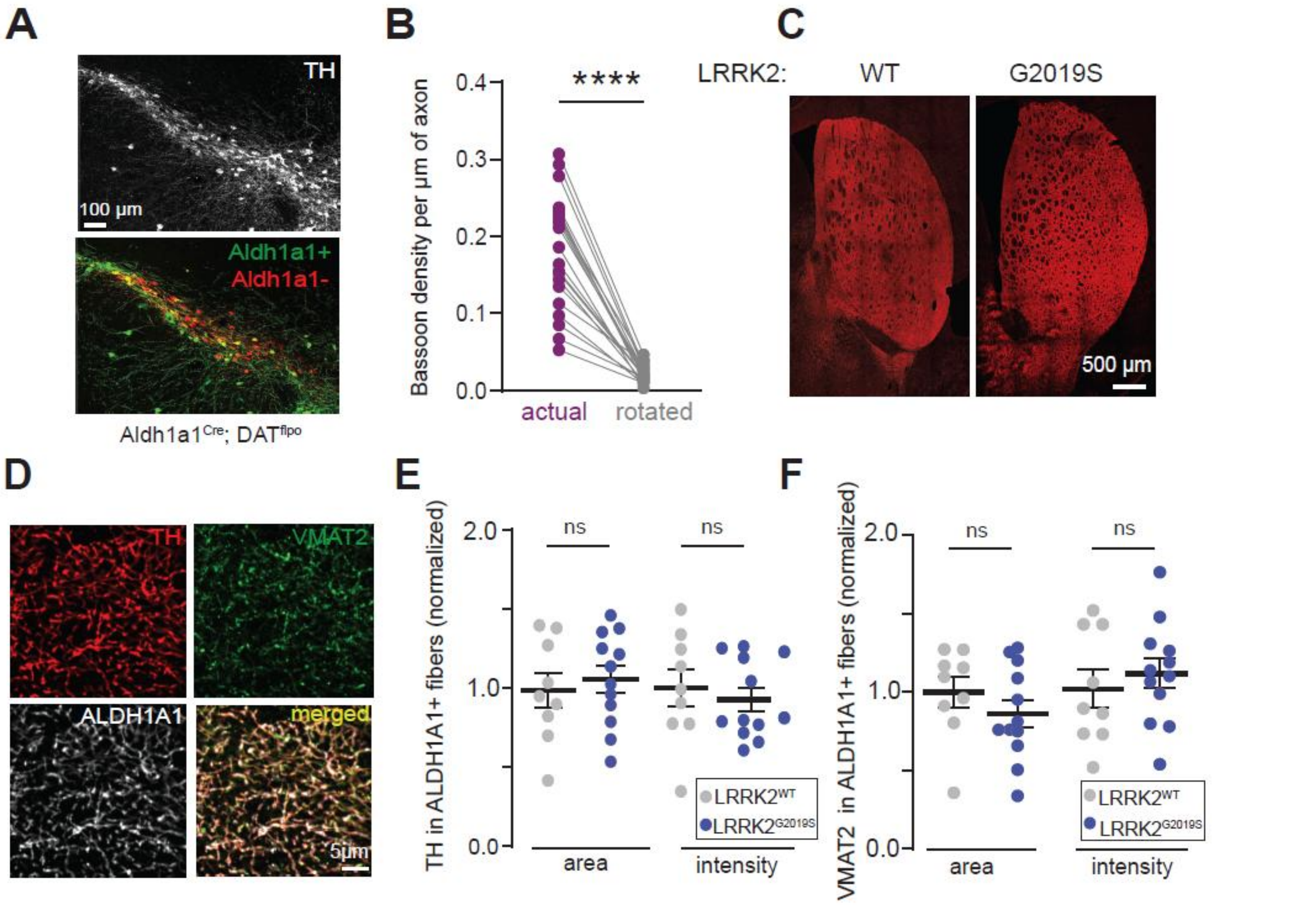

Supplementary Figure 5. LRRK2^{G2019S} changes the number and composition of active zones without affecting overall integrity of TH fibers (linked to Figure 5). A. SNc sections from Aldh1a1^{Cre}; DAT^{flpo} mice injected with viral plasmids stained with EGFP and mCherry to label Aldh1a1+ and Aldh1a1- dopamine neurons, respectively. **B**. Image with bassoon signal in control mice was rotated by 180°, whereas the eGFP channel remained unaltered. Quantification of bassoon density within dopamine axons before and after image rotation. Each circle represents the average result of a region containing 25,000-35,000 bassoon clusters. Data represent mean ±SEM. n=18 (3-6 sections/mouse, 4 mice). Significant differences in comparisons after unpaired t-tests. **** p<0.0001. **C.** Representative low magnification striatal sections from 6-month-old LRRK2^{WT} and LRRK2^{G2019S} mice stained with TH. **D**. Representative high magnification dorsal striatal sections from adult (6 months) LRRK2^{WT} and LRRK2^{G2019S} mice stained with TH, ALDH1A1, and VMAT2 antibodies. **E**. Quantification of TH area and fluorescence intensity in ALDH1A1 immunostained dopamine axons across genotypes in dorsal striatum. **F.** Average VMAT2 area and intensity in TH+/ALDH1A1+ dopamine axons across genotypes in dorsal striatum. For E and F, each dot represents the mean area or intensity of a striatal section, and data show mean±SEM; n=9-12 (2-3sections/mouse, 4-5mice/group). Ns, not significant with unpaired t test**.**

bioRxiv preprint doi: https://doi.org/10.1101/2025.08.28.672006; this version posted August 28, 2025. The copyright holder for this preprint (which was not certified by peer review) is the author/funder, who has granted bioRxiv a license to display the preprint in perpetuity. It is made available under aCC-BY 4.0 International license.

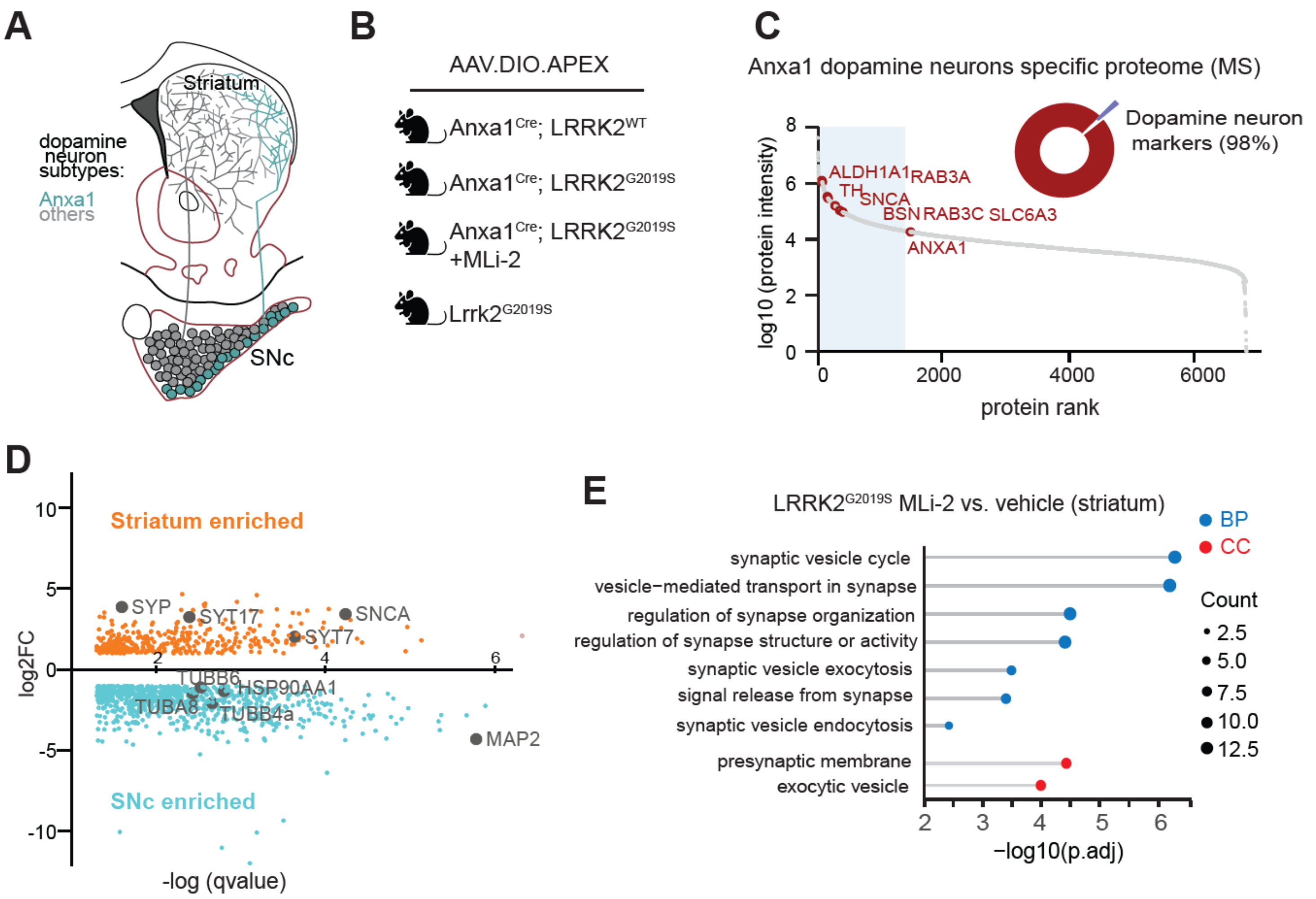

Supplementary Figure 6. Axonal and somatic proteomics of Anxa1+ neurons (linked to Figure 5). A. Schematic illustrating the localization of Anxa1 dopamine neuron subtype in the mouse SNc and their projection patterns in the striatum. **B.** Experiment groups of APEX2-based proximity labeling within genetically labeled dopamine neurons in the mouse brain. **C**. 98% of the top 55 mDA neuron marker genes (e.g., TH and DAT (SLC6A3)) from a publicly available APEX2 proteome dataset (dataset identifier PXD026229 ProteomeXchange Consortium) were detected in all our mass spectrometry samples from DAT^{Cre}; APEX2 EGFP mice. **D.** Differential expression comparison of SNc and Striatum APEX+ streptavidin pulldown samples. Proteins colored orange or blue indicated significantly enriched in Striatum vs. SNc, respectively. ($|Log2FC| > 0.58$ and unadjusted p-value ≤ 0.05 by multiple unpaired t-tests) **E.** Gene Ontology analysis of proteins with at least one differentially regulated phosphopeptide in striatum from vehicle vs. MLi2-treated Anxa1iCre $LRRK2^{G2019S}$ mice. BP, Biological Process; CC, Cellular Component refer to categories in GO. All enriched pathways have been uploaded to Zenodo; the link can be found in the key resources table.

bioRxiv preprint doi: https://doi.org/10.1101/2025.08.28.672006; this version posted August 28, 2025. The copyright holder for this preprint (which was not certified by peer review) is the author/funder, who has granted bioRxiv a license to display the preprint in perpetuity. It is made available under aCC-BY 4.0 International license.

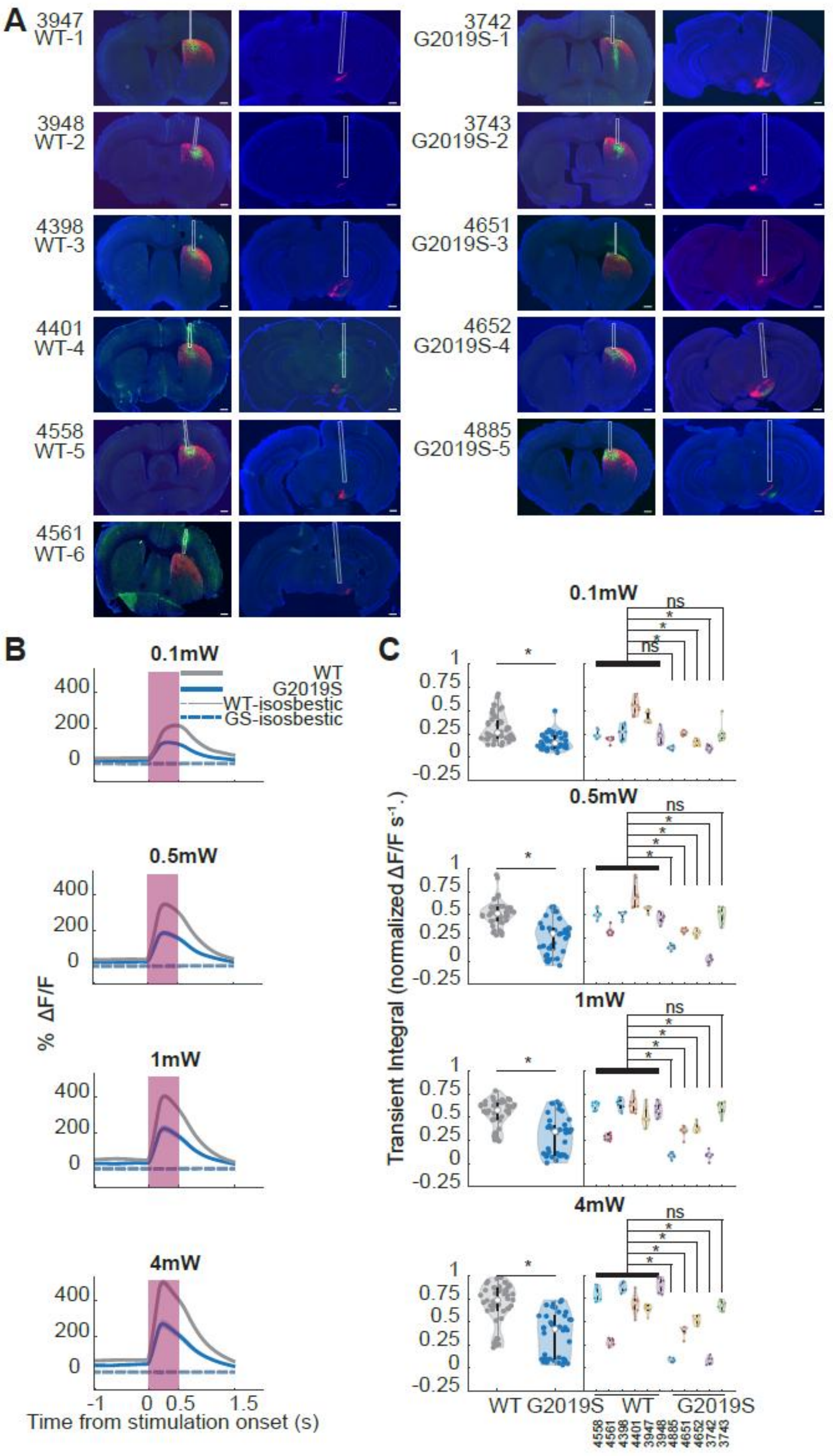

Supplementary Figure 7. Histology and power dependence for in vivo evoked dopamine measurements (linked to Figure 6).

A. Histological verification of viral expression and fiber placement in each animal. Left two columns, coronal sections of the dorsal striatum region (dStr) and the Substantia Nigra pars compacta (SNc) of each LRRK2^{WT} mouse used in the analysis. Red fluorescence indicates the expression of ChRmine in the somas (SNc) and the axons (dStr) of Anxa1+ dopaminergic neurons. Green fluorescence indicates the expression of GRAB-DA3m in the dorsal striatum. White rectangles indicate where the photometry (dStr) and optogenetics (SNc) fibers were placed. Right two columns, same as

bioRxiv preprint doi: https://doi.org/10.1101/2025.08.28.672006; this version posted August 28, 2025. The copyright holder for this preprint (which was not certified by peer review) is the author/funder, who has granted bioRxiv a license to display the preprint in perpetuity. It is made available under aCC-BY 4.0 International license.

left, but in LRRK2^{G2019S} mice. Scale bar = 0.5 mm. **B.** Same as Figure 6C but with 0.1 mW, 0.5 mW, 1 mW, and 4 mW optogenetic light powers from top row to bottom row (blue trace, LRRK2^{G2019S} mice = 5, n = 40 stimulations; grey trace, LRRK2^{WT} mice = 6, n = 48 stimulations). Shaded regions denote mean ± SEM across stimulations. **C.** Same as Figure 6D but with 0.1 mW, 0.5 mW, 1 mW, and 4 mW optogenetic light powers from top row to bottom row. White dots denote group means and black bars denote 25th to 75th percentile. Left, group comparisons at each power (LRRK2^{G2019S} n = 40 stimulations per power, LRRK2^{WT} n = 48 stimulations per power; p-value (0.1 mW) = 4.17E-7, p-value (0.5 mW) = 2.01E-11, p-value (1 mW) = 1.97E-9, p-value (4 mW) = 7.02E-10, unpaired t-test). Right, dopamine release response in individual mice. Numbers denote mouse ID. Asterisks (*) on the right denote p-value < 0.01 from Welch's t-test with Bonferroni correction ($\alpha = 0.05/5 = 0.01$) comparing individual LRRK2^{G2019S} mouse to the LRRK2^{WT} group.

bioRxiv preprint doi: https://doi.org/10.1101/2025.08.28.672006; this version posted August 28, 2025. The copyright holder for this preprint (which was not certified by peer review) is the author/funder, who has granted bioRxiv a license to display the preprint in perpetuity. It is made available under aCC-BY 4.0 International license.

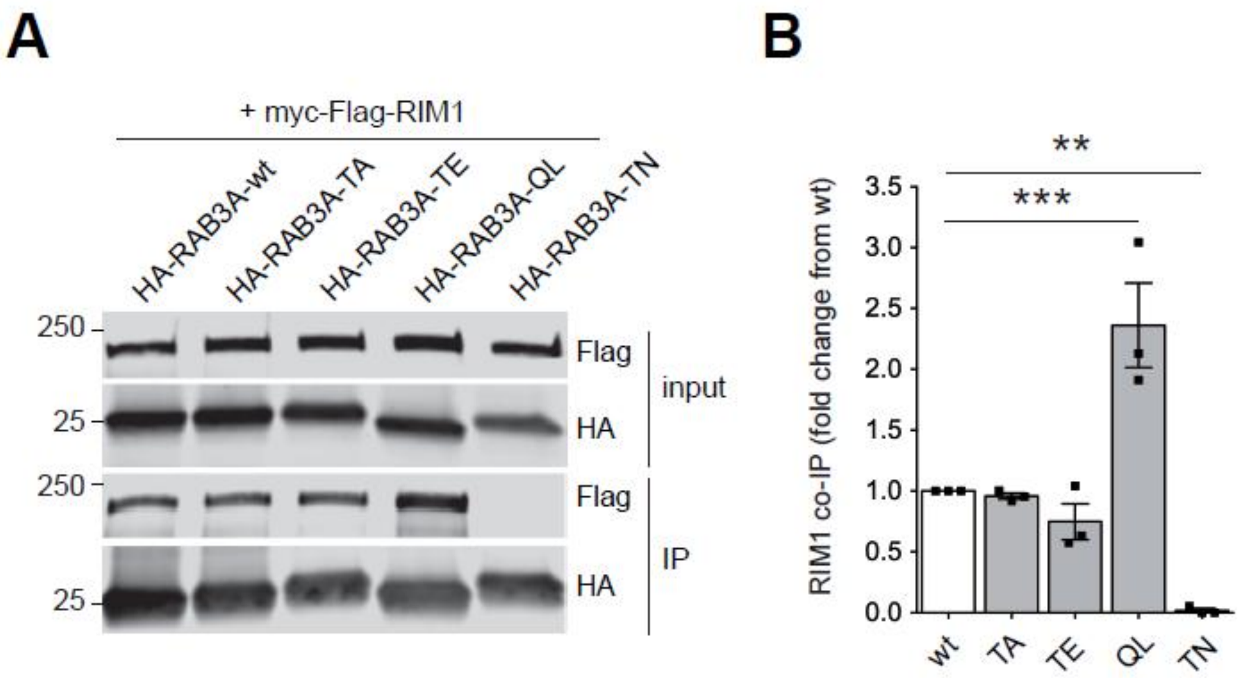

Supplementary Figure 8. Phosphomimetic RAB3 constructs fail to reproduce the effects of phosphorylated RAB3 proteins (linked to Figure 3).
A. HEK293 cells were transiently co-transfected with HA-tagged RAB3A constructs and myc-flag-RIM1. Extracts were subjected to pulldown with anti-HA beads, and input and immunoprecipitated material (IP) subjected to blotting with the indicated antibodies. WT, phospho-deficient (RAB3A-T86A) and phospho-mimic (RAB3A-T86E) RAB3A show similar binding to tagged RIM1. In contrast, and as expected, the RAB3A mutant mimicking the GTP-bound state (Q81L) shows increased RIM1 binding, whilst the RAB3A mutant mimicking the GDP-bound state (T36N) shows loss of RIM1 binding.
B. Quantification of experiments of the type shown in A. Co-immunoprecipitation of myc-flag-RIM1 with the indicated HA-tagged RAB3A constructs was normalized to that obtained with wild-type (wt) RAB3A. n=3 independent experiments (mean±SEM). Asterisk indicates statistical significance as determined by one-way ANOVA with Dunnett's multiple comparisons. ***p<0.001; **p<0.0.
